# *Candida* spp. with Acquired Echinocandin Resistance, France, 2004–2010^1^

**DOI:** 10.3201/eid1801.110556

**Published:** 2012-01

**Authors:** Eric Dannaoui, Marie Desnos-Ollivier, Dea Garcia-Hermoso, Fredéric Grenouillet, Sophie Cassaing, Marie-Thérèse Baixench, Stéphane Bretagne, Françoise Dromer, Olivier Lortholary, the French Mycoses Study Group

**Affiliations:** Institut Pasteur, Paris, France (E. Dannaoui, M. Desnos-Ollivier, D. Garcia-Hermoso, S. Bretagne, F. Dromer, O. Lortholary);; Centre Hospitalier Universitaire de Besançon, Besançon France (F. Grenouillet);; Centre Hospitalier Universitaire de Toulouse–Hôpital Rangueil, Toulouse, France (S. Cassaing);; Hôpital Cochin, Paris (M.-T. Baixench)

**Keywords:** caspofungin, echinocandins, FKS mutation, Candida albicans, Candida glabrata, Candida krusei, treatment failure, clinical isolates, genotyping, drug resistance, fungi, France

## Abstract

We report 20 episodes of infection caused by acquired echinocandin-resistant *Candida* spp. harboring diverse and new Fksp mutations. For 12 patients, initial isolates (low MIC, wild-type Fksp sequence) and subsequent isolates (after caspofungin treatment, high MIC, mutated Fksp) were genetically related.

Echinocandins are effective in patients with invasive candidiasis and recommended as first-line therapy, especially for patients with severe sepsis or those previously exposed to azoles or infected with *Candida glabrata* (*1*). Fewer than 50 persons infected with echinocandin-resistant species that are usually susceptible, such as *C. albicans*, *C. glabrata*, *C. tropicalis*, and *C. krusei*, have been described in limited series or case reports (*2**–**4*). All species were found in patients preexposed to echinocandins. The major mechanism of resistance is related to mutations in *FKS* genes coding for β-1,3-glucan-synthase (*5*), with almost 20 known *FKS* mutations. We describe the characteristics of infections from caspofungin-resistant *Candida* spp. isolates belonging to usually susceptible species recorded in France (2004–2010) and analyze their *FKS* mutations and effect on echinocandin susceptibility.

## The Study

Isolates received at the French National Reference Center for Mycoses and Antifungals (NRCMA) are identified to the species level by standard mycologic procedures and routinely tested for susceptibility to caspofungin, micafungin, and anidulafungin by using European Committee for Antimicrobial Susceptibility Testing (EUCAST) methods (*6*) and AM3 medium (*7*). In addition, RPMI 1640 medium was used here for selected isolates and reference strains. For the clinical isolates with caspofungin MIC >0.5 µg/mL in AM3, nucleotide sequences of hot spot (HS) 1 and 2 regions of the *FKS1* gene for *C. albicans* and *C. krusei* and of HS1 region of *FKS1*, *FKS2*, and *FKS3* genes for *C. glabrata* were determined (*7**,**8*).

The resulting protein sequences were aligned with the BioloMics software (BioloMics, BioAware SA, Hannut, Belgium) and compared with reference strains (*C. albicans*, ATCC32354; *C. krusei*, ATCC6258; and *C. glabrata*, ATCC2001). Genetic relatedness of *C. albicans* and *C. glabrata* paired isolates was studied by using microsatellite-length polymorphism analysis (*9**–**11*). The Wilcoxon signed-rank test was used to compare echinocandin MICs of paired isolates. Surveillance for mycoses by the NRCMA has been approved by the Institut Pasteur Internal Review Board and by the Commission Nationale de l’Informatique et des Libertés.

During September 2004–April 2010, twenty proven infections caused by *C. albicans* (n = 10), *C. glabrata* (n = 8), or *C. krusei* (n = 2) with caspofungin MIC >0.5 µg/mL and a mutation in the target enzyme were reported to the NRCMA (Table 1). Nineteen of the isolates were recovered after caspofungin treatment for a median duration of 27 days (range 10–270 days; 13 of 19 patients received caspofungin at the time the resistant isolate was recovered). Caspofungin was prescribed for 14 patients with proven *Candida* spp. infection, 1 patient with proven invasive aspergillosis, and 2 patients with febrile neutropenia; for 2 persons with hematologic malignancies, caspofungin was prescribed prophylactically.

**Table 1:** Characteristics of 20 patients with infections caused by a non–*parapsilosis/guilliermondii Candida* spp. Fks mutation, France, 2004–2010*

Patient no.	Age, y/sex	Underlying condition	Neutropenia	Species	Site of infection	Duration of caspofungin exposure, d†	Outcome at 30 d‡
1	34/M	HIV positive	No	*C. albicans*	Esophagus	21	Alive
2	20/M	Hematologic malignancy: familial lymphohistiocytosis	Yes	*C. albicans*	Blood	17	Dead
3	77/M	Hematologic malignancy: AML	Yes	*C. albicans*	Blood	25	Alive
4	46/M	Hematologic malignancy: AML	Yes	*C. albicans*	Blood, peritoneum, pleural fluid	26	Dead
5	34/F	Liver transplant: cirrhosis	No	*C. albicans*	Hepatic abscess, peritoneum	60	Alive
6	64/F	Hematologic malignancy: AML; breast cancer	No	*C. albicans*	Blood	25	Alive at 17 d
7	59/M	Teratocarcinoma	No	*C. albicans*	Pharynx	35	Dead
8	28/M	Chronic mucocutaneous candidiasis	No	*C. albicans*	Pharynx, nails	270	Alive
9	14/F	Hematologic malignancy: ALL	Yes	*C. krusei*	Lung	45	Alive
10	79/M	Hematologic malignancy: non-Hodgkin lymphoma	Yes	*C. krusei*	Blood	10	Dead
11	46/M	Hematologic malignancy: Burkitt lymphoma; HSCT	Yes	*C. glabrata*	Blood	None	Dead
12	85/M	Gastric ulcer; CVC	No	*C. glabrata*	Blood	32	Alive
13	28/M	Hematologic malignancy: non-Hodgkin lymphoma; HSCT	No	*C. glabrata*	Palate§	135	Alive
14	48/M	Esophageal cancer	No	*C. glabrata*	Blood	12	Alive
15	41/M	Liver transplant: fulminant hepatitis	No	*C. glabrata*	Blood, peritoneum	37	Dead
16	38/F	Hematologic malignancy; AML; HSCT	Yes	*C. glabrata*	Blood	51	Dead
17	60/M	Acute pancreatitis; GI tract surgery	No	*C. glabrata*	Bile	34	Alive
18	39/M	Hematologic malignancy: AML; HSCT	No	*C. glabrata*	Sinus§	15	Alive
19	55/F	Lock-in syndrome; neurogenic bladder	No	*C. glabrata*	Urine¶	27	Alive
20	63/M	Colon cancer	Yes	*C. glabrata*	Blood	14	Alive

*AML, acute myelogenous leukemia; ALL, acute lymphoblastic leukemia; HSCT, hematopoietic stem cell transplantation; CVC, central venous catheter; GI, gastrointestinal. †Duration of caspofungin exposure before isolation of the first resistant *Candida* isolate. ‡Outcome 30 d after isolation of the first resistant *Candida* isolate. §From a biopsy specimen. ¶With sepsis.

The geometric mean MIC for *C. glabrata* and *C. albicans* were 2.8 and 1.7 µg/mL for caspofungin, 0.4 and 0.7 µg/mL for micafungin, and 0.2 and 0.09 µg/mL for anidulafungin, respectively (Table 2). Of the 20 mutated isolates found resistant to caspofungin in AM3 by using the EUCAST method, 19 also were resistant to caspofungin (1 intermediate), 18 to micafungin (1 intermediate and 1 susceptible), and 9 to anidulafungin (5 intermediate and 6 susceptible) according to Clinical Laboratory Standards Institute (CLSI) breakpoints and RPMI 1640 medium (Table 2). According to EUCAST breakpoints, 19 isolates also were resistant to anidulafungin, and 1 isolate was almost resistant (MIC 0.03 µg/mL). We thus showed discrepancies between CLSI and EUCAST regarding anidulafungin susceptibility (www.srga.org/eucastwt/MICTAB/EUCAST%20clinical%20MIC%20breakpoints%20-%20antimicrobials%20for%20Candida%20infections.htm [V 3.0 2011–4-27]) (*12*,*13*).

**Table 2:** In vitro susceptibility and Fksp mutations of 20 echinocandin-resistant *Candida* spp. isolates, France, 2004–2010

Patient no.	Strain	Species	MIC, µg/m, AM3/RPMI 1640 medium				Fksp mutation	
Caspofungin	Micafungin	Anidulafungin	Gene	Mutation
1*	05BL1-38	*C. albicans*	1/2	0.25/1	0.06/0.125		*FKS1* (HS1)	F641S
2*	ODL13-1254	*C. albicans*	1/2	1/1	0.5/0.5		*FKS1* (HS1)	S645Y
3†	06BL2-127	*C. albicans*	2/2	1/0.5	0.125/0.125		*FKS1* (HS1)	F641S‡ + S645P‡
4	ODL19-1894	*C. albicans*	4/2	2/2	0.125/0.25		*FKS1* (HS1)	S645P
5*	08BL1-94	*C. albicans*	2/4	0.25/1	0.06/0.5		*FKS1* (HS2)	R1361G§
6*	08BL2-143	*C. albicans*	8/4	4/2	0.25/0.5		*FKS1* (HS1)	S645P
7*	09BL1-43	*C. albicans*	1/2	0.25/1	0.06/0.25		*FKS1* (HS1)	F641S
8*	09BL1-77	*C. albicans*	0.5/0.5	0.5/0.25	0.015/0.03		*FKS1* (HS1)	R647G§
9	06BL1-34	*C. krusei*	4/8	2/4	1/2		*FKS1* (HS1)	L648W§,¶
10*	10BL1-50	*C. krusei*	2/4	1/2	0.06/1		*FKS1* (HS1)	F645L§,¶
11	ODL7-647	*C. glabrata*	8/8	0.5/1	0.25/0.125		*FKS2*	DelF658#
12*	07BL2-157	*C. glabrata*	4/1	1/0.5	0.25/0.5		*FKS2*	DelF658#
13*	06BL1-33	*C. glabrata*	8/8	4/8	2/2		*FKS2*	S663P
14*	ODL21-2028	*C. glabrata*	1/1	0.25/0.25	0.25/0.25		*FKS1*	S629P
15*	ODL22-2183	*C. glabrata*	8/2	0.25/0.25	0.25/1		*FKS2*	S663P
16	ODL23-2221	*C. glabrata*	1/4	0.06/2	0.06/0.25		*FKS1* + *FKS2*	F625I§ (*FKS1*) + P667T§ (*FKS2*)
17*	08BL2-142	*C. glabrata*	1/4	0.25/2	0.25/2		*FKS2*	S663P
18	09BL1-55	*C. glabrata*	8/4	2/4	0.5/0.5		*FKS2*	S663P
19	10BL1-19	*C. glabrata*	0.5/4	0.06/0.5	0.06/1		*FKS2*	F659S + L664V§
20	10BL1-67	*C. glabrata*	4/4	0.5/1	0.125/1		*FKS2*	DelF658#

*Parentage of initial isolate available. †In this patient, another isolate with reduced susceptibility to echinocandin was retrieved. This isolate harbored an S645P mutation in *FKS1*. ‡Heterozygous mutation. §Mutations not already described (*13*). ¶Strains had also an L701M mutation. #Deletion.

Of the 10 caspofungin-resistant *C. glabrata* isolates, 8 harbored a mutation in Fks2p only, 1 isolate had a mutation in Fks1p, and 1 had mutations in Fks1p and Fks2p (Table 2). Of the 8 caspofungin-resistant *C. albicans* isolates, 1 had a missense mutation in HS2, and 1 had a combination of 2 heterozygous mutations in HS1. The other 6 isolates harbored 4 different mutations in HS1 (Table 2). Finally, the 2 *C. krusei* isolates had 2 different mutations in HS1 region. Of the 20 mutated isolates, 6 harbored 7 mutations not yet described in the literature (Table 2) (*13*).

Prior initial isolates available for 12 patients had the wild-type sequence for the HS regions that were mutated in the paired resistant isolate. All initial isolates were susceptible to anidulafungin and to micafungin and anidulafungin according to EUCAST and CLSI, respectively (data not shown). According to CLSI caspofungin breakpoints, 5 of 6 initial isolates of *C. albicans* were susceptible, and 1 was intermediate; 4 of 5 *C. glabrata* isolates were resistant (0.5 µg/mL), and 1 was intermediate; and the *C. krusei* isolate was resistant (1 µg/mL). For each of the 12 pairs, MICs increased significantly (from 3 to 8 dilutions for caspofungin and micafungin and from 1 to 8 dilutions for anidulafungin) between the wild-type and the mutant isolate (Figure; p<0.001). Genetic relatedness was demonstrated for all *C. albicans* and *C. glabrata* paired isolates.

**Figure:**
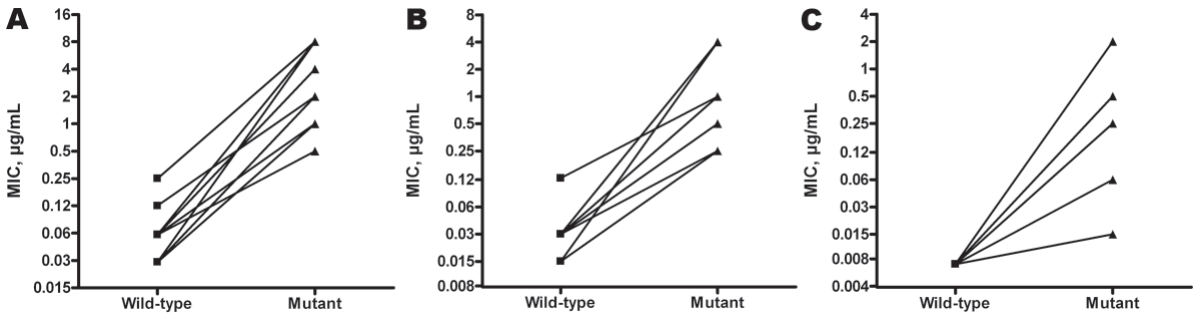
Corresponding caspofungin (A), micafungin (B), and anidulafungin (C) MICs in 12 Fksp mutant *Candida* spp. isolates and their wild-type parent isolates, France, 2004–2010. Susceptibility testing was performed by using the European Committee for Antimicrobial Susceptibility Testing method (*6*) and AM3 medium (*7*).

## Conclusions

We demonstrated that recent exposure to caspofungin altered the distribution of species causing *Candida* bloodstream infections (*14*), and that caspofungin exposure was independently associated with fungemia associated with intrinsically less-susceptible species in hematology (*15*). Echinocandin resistance in *Candida* spp. is still uncommon (*4**,**13*). Through our surveillance program, we estimated the incidence of decreased susceptibility to caspofungin associated with *FKS* mutations among *C. albicans, C. glabrata*, and *C. krusei* isolates responsible for candidemia in children and adults in Paris at 6 (0.4%) of 1,643 (NRCMA, unpub. data). We report proven caspofungin-resistant *Candida* spp. infections with none of the isolates belonging to the intrinsically less-susceptible species *C. parapsilosis* or *C. guilliermondii*.

We determined antifungal susceptibility testing by the EUCAST technique using AM3 because it enables better discrimination between susceptible wild-type and resistant mutant isolates (*7*). All isolates with high caspofungin MIC (>0.5 µg/mL) had mutation in the HS1 and/or HS2 region of *FKS* genes. The mutations were not restricted to a given position but were diverse, especially for *C. albicans* with 6 different mutations among the 8 resistant isolates; 5 different mutations were observed among the 10 *C. glabrata* resistant isolates. Most mutations in *C. glabrata* isolates were in Fks2p. Two mutations in *C. albicans*, 2 patterns of mutation in *C. glabrata*, and 1 mutation in *C. krusei* had not been reported before, highlighting the great mutation diversity that could be responsible for echinocandin resistance (*13*).

All but 1 patient had received caspofungin (70 mg on day 1, then 50 mg/d) before recovery of the resistant isolate, with a variable duration of exposure (<10 days to >8 months), in agreement with the literature (5 [*3*] to 420 days). In addition, 13 of 19 patients received caspofungin at the time of recovery of the resistant isolate. Most patients had malignancy, but 7 intensive care unit hospitalizations also were recorded. Echinocandins MICs between the wild-type parent and the subsequent mutant isolate increased by up to 8 log_2_ dilutions (Figure). The source of the resistant isolate is not unequivocal; it was acquired from the environment as an already resistant isolate or from the patient’s own flora under drug pressure. Our genotyping results favor the second hypothesis. This study suggests in France the emergence of infections from acquired echinocandin resistance in usually susceptible *Candida* spp. in patients preexposed to caspofungin, which highlights the need for careful species identification, antifungal drug susceptibility testing, and evaluation of prior drug exposure before antifungal drug prescription.
